# Serial Molecular‐Microstructural Multimodal Intravascular Imaging of Atherosclerotic Plaques in Mice

**DOI:** 10.1002/advs.77316

**Published:** 2026-08-19

**Authors:** Jiawen Li, Joanne T. M. Tan, Lei Xiang, Jiandi Liu, Ayla Hoogendoorn, Victoria A. Nankivell, Lauren Sandeman, Richard Le, Rouyan Chen, Robert A. McLaughlin, Peter J. Psaltis, Johan W. Verjans, Christina A. Bursill

**Affiliations:** ^1^ Discipline of Biomedical Engineering School of Electrical and Mechanical Engineering Adelaide University Adelaide South Australia Australia; ^2^ Institute for Photonics and Advanced Sensing Adelaide University Adelaide South Australia Australia; ^3^ Australian Research Council (ARC) Centre of Excellence for Nanoscale BioPhotonics Adelaide South Australia Australia; ^4^ Vascular Research Centre Lifelong Health Theme South Australian Health and Medical Research Institute (SAHMRI) Adelaide South Australia Australia; ^5^ Adelaide Medical School Adelaide University Adelaide South Australia Australia; ^6^ Department of Cardiology Second Affiliated Hospital Jiangxi Medical College Nanchang University Nanchang Jiangxi China; ^7^ Flinders Medical Centre Adelaide South Australia Australia; ^8^ School of Biomedicine Adelaide University Adelaide South Australia Australia; ^9^ Department of Cardiology Royal Adelaide Hospital Adelaide South Australia Australia

**Keywords:** fluorescence, intravascular imaging, murine models, optical coherence tomography, plaque evolution

## Abstract

Murine models are essential for cardiovascular translational research for developing new therapies to reduce the risk of heart attacks. However, assessment of murine atherosclerotic plaques by traditional histological methods fails to capture the entirety of plaque development across the length of a blood vessel over time. Here, the authors have developed and validated the first longitudinal molecular and microstructural intravascular imaging approach to study murine atherosclerosis. This approach combines a unique murine surgical model and a miniaturized multimodal intravascular imaging device with optical coherence tomography (OCT) and fluorescence imaging capabilities able to detect infused indocyanine green (ICG), a marker of macrophages. This multimodal device could identify important plaque features and their changes over multiple time points across the length of vessels containing atherosclerosis in high‐cholesterol diet‐fed apolipoprotein E knockout mice. It is found that OCT‐derived plaque lipid measures correlate closely with histologically assessed plaque lipid (Oil Red O) in co‐registered sections. Plaque ICG fluorescence also correlates with CD68+ macrophages, measured by immunofluorescence in co‐registered sections. This novel approach maximizes data acquisition of plaque evolution dynamics within individual vessels. It represents a paradigm shift over the current need to compare disparate atherosclerosis plaques histologically across multiple timepoints and in multiple animals.

## Graphical abstract

A novel longitudinal molecular and microstructural intravascular imaging approach is developed to study murine atherosclerosis. The technique combines a unique surgical model with a miniaturized multimodal device to detect macrophage activity and lipid simultaneously. This approach enables the tracking of atherosclerotic plaque features across the entire length of vessels over time, revealing strong correlations between imaging metrics and histology.

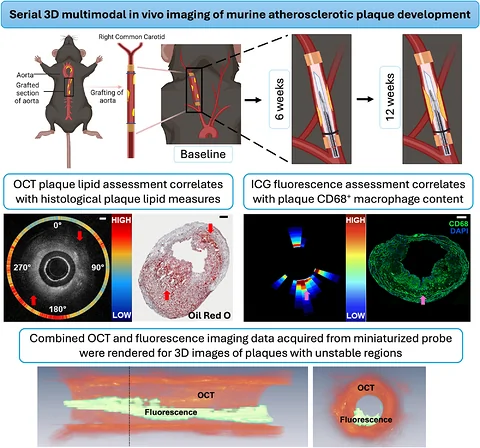

## 1 Introduction

Atherosclerotic coronary artery disease is the most common cause of death in middle‐ and high‐income countries worldwide [1]. Many atherosclerotic plaques grow and remain stable, while others destabilize and rupture, causing myocardial infarction (commonly known as heart attack) [2]. The ability to capture the evolution of a stable plaque into a high‐risk plaque would be immensely valuable and impact clinical approaches to treating coronary artery disease [3, 4, 5, 6].

Murine models are essential to study atherosclerosis systematically by presenting the opportunity for genetic knock‐out/overexpression of a certain gene or labelling with fluorescence [7, 8, 9]. Currently, measuring atherosclerotic plaque development at multiple time points in murine models mainly relies on histological assessment in separate cohorts of mice due to the need to terminate animal life [10]. This introduces inter‐cohort data variability and requires more animals with large sample sizes to ensure statistical power.

Our newly developed miniaturized multimodal imaging device that combines near‐infrared fluorescence (NIRF) with optical coherence tomography (OCT) has the potential to overcome these limitations. It provides simultaneous high‐resolution structural and molecular information from atherosclerotic plaques [11, 12, 13, 14]. Building on this, we have also developed a novel murine surgical model that enables the serial imaging of atherosclerotic vessels when combined with our miniaturized multimodal intravascular imaging probe. This murine model incorporates carotid interposition grafting of the descending thoracic aorta, which has a larger vessel diameter than the carotid [15]. Our miniaturized catheter (0.635 mm, i.e., 1.9 Fr, compared to the clinically used OCT catheter at 2.7 Fr) can be inserted into the thoracic aorta that is grafted into the carotid region [16, 17]. During the imaging process, under anesthesia, the carotid region is exposed, the grafted vessel is clamped at two sites, and a transverse arteriotomy is made to allow catheter insertion. When conducted in atherosclerosis‐prone apolipoprotein (*Apo*)*e* ^−/−^ mice, this unique combination of our surgical murine model and miniaturized imaging probe enables longitudinal intravascular visualization and monitoring of atherosclerotic plaque development over multiple timepoints within the same animal, exemplifying a true serial imaging approach.

Accordingly, in *Apoe* ^−/−^ mice fed a high‐cholesterol diet (HCD), we evaluated the ability of our miniaturized OCT+fluorescence imaging device to identify important plaque features and their changes over time in the murine carotid interposition grafting model. Vessel lipid measurement was achieved using an OCT attenuation algorithm, while detection of plaque macrophages was based on fluorescence derived from the NIRF dye indocyanine green (ICG). Our analyses revealed a compelling correlation between plaque features, captured by our dual‐modality imaging probe, and systematic *ex vivo* plaque data assessed histologically in co‐registered sections. By combining murine surgical model and intravascular imaging innovations, this study demonstrates the ability to capture intravascular imaging of plaque volume longitudinally, combined with the distribution of inflammation throughout the plaque in three dimensions. This platform offers a powerful, more accurate approach to testing new therapies or determining the effects of genetic deletions on plaque development and composition over time, facilitating future clinical translation.

## 2 Results

### 2.1 Serial Imaging of Atherosclerotic Plaque by Intravascular OCT+Fluorescence Device

We performed serial imaging of atherosclerotic plaque development in a mouse model, utilizing an established carotid interposition grafting model [15]. The thoracic aortas from donor mice were interposition‐grafted into the right common carotid arteries of recipient mice (Figure 1A). This allowed us to image the largest artery (thoracic aorta) in a mouse with an inner diameter of 1.2–1.3 mm, similar to a mid‐distal end of a human coronary artery. Placing the thoracic aorta in the carotid region enables longitudinal assessments, as clamping of the right carotid artery can be performed for the imaging period, without causing death, as blood supply is maintained to the brain via the neighboring left carotid artery. This was a critical element to allow serial intravascular imaging at two timepoints postgrafting surgery. Baseline OCT images of thoracic aortas were acquired at the time of grafting surgery. As the thoracic aortas were from donor mice fed a HCD for 6 months, characteristic features of established atherosclerotic plaques were evident including cholesterol crystals (labelled CC, denoted by a yellow arrow at 7 o'clock, Figure 1B), loss of layered intima, media and adventitia structure (at 5–10 o'clock, Figure 1B) and significant intimal thickening [18]. We then tracked compositional and structural changes over time by imaging the grafted aorta in the recipient mice at weeks 6 (Figure 1C) and 12 (Figure 1D) postsurgery. The morphological plaque features captured by our OCT imaging probe were well‐matched with co‐registered histological H&E‐stained sections (Figure 1E).

**FIGURE 1:**
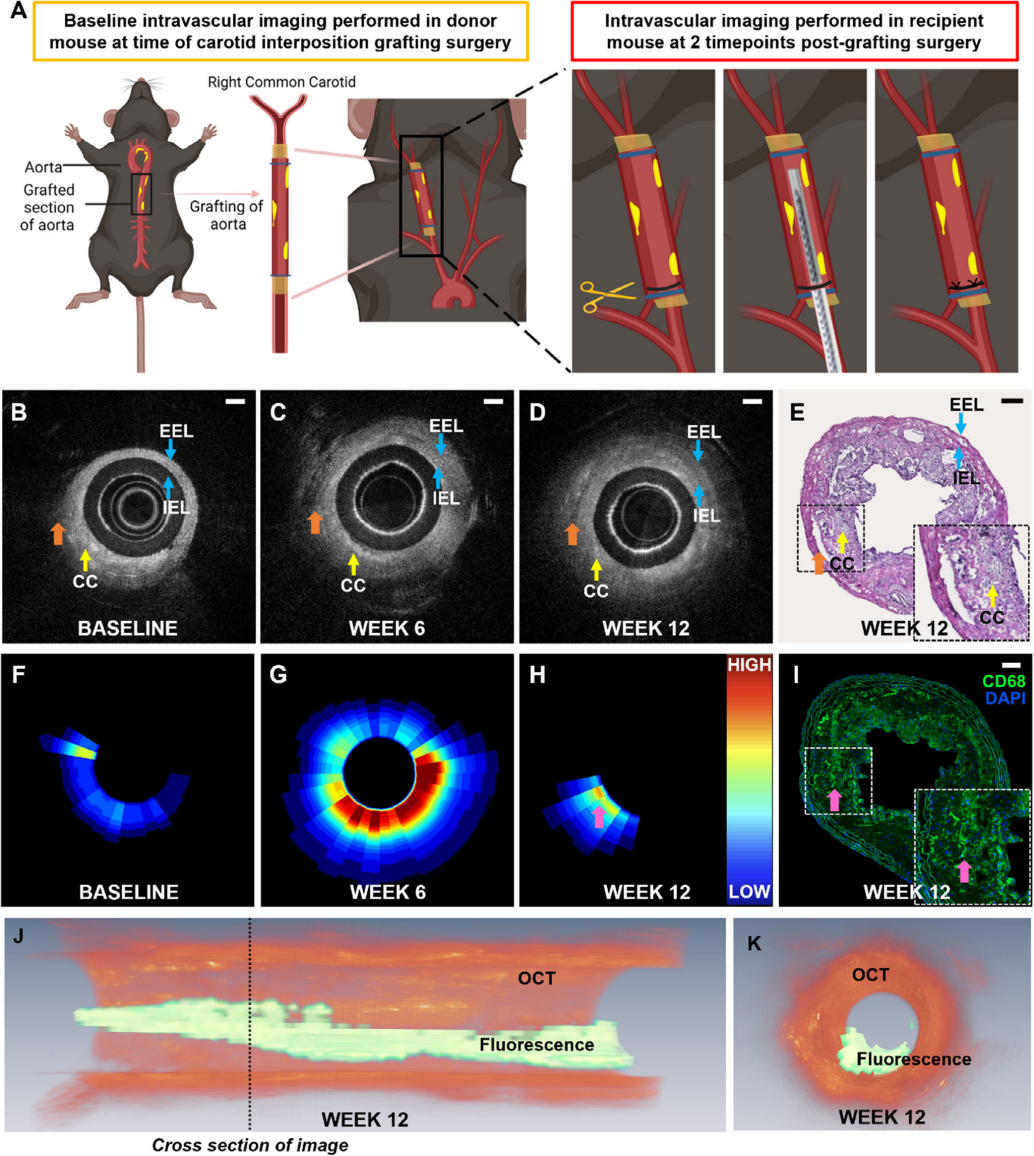
Serial imaging of atherosclerotic plaque by intravascular OCT+fluorescence device. (A) Murine carotid interposition grafting surgical model. *Apoe^−/−^* donor mice were fed HCD for 6 months. The thoracic aorta from the donor mouse was carotid interposition‐grafted into *Apoe^−/−^* recipient mice fed HCD for 3 months prior to surgery. In vivo intravascular imaging of the grafted aorta was performed at surgery (baseline) and then 6‐ and 12‐weeks postsurgery (*n* = 4). Representative OCT images at (B) baseline, (C) Week 6 and (D) Week 12 postsurgery. Orange arrow pointing to a structural feature used for matching images of the three time points together with cholesterol crystals (CC, yellow arrow), internal elastic lamina (IEL) and external elastic lamina (EEL). (E) Corresponding H&E image of cross section from Week 12 timepoint. Square inset shows a magnified section of image. Fluorescence images taken simultaneously at (F) baseline, (G) Week 6 and (H) Week 12 postgrafting. Pseudo‐colored fluorescent intensity scale showing high (red) to low (blue) ICG fluorescence. (I) Corresponding cross‐sectional image of immunofluorescently stained CD68^+^ macrophages (green staining, pink arrow) from Week 12 timepoint. Square inset shows a magnified section of the image. (J) 3D reconstruction of OCT+fluorescence images of the entire artery obtained at the Week 12 timepoint. Red channel: OCT; light green channel: ICG fluorescence. The radial axis of the fluorescence images (F, G, H) represents wavelength, not tissue depth, and provides a color‐coded representation of the spectrum acquired at each orientation. The black line indicates positioning of the cross section of the artery (K) that corresponds to E and I ex vivo histological images. Scale bars: 100 µm.

The dual modality of our miniaturized imaging catheter allowed us to collect simultaneous OCT and NIRF images (Figure 1F–H). We previously showed that ICG targets macrophages in areas of enhanced endothelial permeability in carotid plaque specimens [14]. Similar results have been shown in vivo in rabbits and pigs with OCT‐NIRF imaging catheters [19, 20] and with human coronary plaques by using combined NIRF and intravascular ultrasound catheters [21]. As indicated by the pseudo‐colored fluorescent intensity scale, the strongest (red) ICG fluorescence was observed at the week 6 middle timepoint (Figure 1G), compared to baseline (Figure 1F). The ICG fluorescence signal then became weaker at the final week 12 imaging timepoint (blue and yellow, Figure 1H), compared to the middle timepoint. Areas with high fluorescence intensity, as captured by intravascular imaging (Figure 1H), were consistent with areas of histologically determined CD68^+^ plaque macrophages (Figure 1I). 3D reconstruction of the acquired imaging data allowed visualization of the plaque across the entire vessel. This revealed a heterogeneous distribution of plaque along the mouse artery that included both structural features, as detected by OCT, and cellular composition by NIRF (Figure 1J,K and Movie S1).

We found reductions in histologically measured macrophages and imaging‐derived fluorescence measures from the 6‐week timepoint to the final 12‐week timepoint. This decrease is expected as more established plaques are less dynamic and fewer monocytes and macrophages are recruited. Furthermore, established plaques have a high proportion of macrophages that are in an apoptotic/necrotic state. Extracellular lipid and cellular debris therefore start to predominate within the plaque, which cannot be detected by macrophage immunofluorescence, and ICG will not co‐locate in these regions.

To further validate this novel model, the specific effects of the intravascular imaging procedure on the vasculature and plaque were assessed. To do this, we included a nonimaged control cohort in which mice underwent the same surgical grafting procedure and HCD feeding but were not subjected to intravascular imaging. At study endpoint, sections spanning vessels from both groups were analyzed histologically for endothelial and medial integrity, markers of inflammation, and plaque area. We found no differences in CD31+ endothelial cells between vessels subjected to the intravascular imaging procedure and those that were not (Figure S1A), indicating minimal endothelial damage associated with the imaging procedure. Furthermore, there were no differences between groups in αSMA+ smooth muscle cells (Figure S1B), indicating the imaging procedure does not compromise medial structural integrity or plaque stability. Assessment of inflammatory mediators revealed there were no differences in vascular cell adhesion molecule 1 (VCAM1, Figure S1C), but inducible nitric oxide synthase (iNOS) and the chemokine CCL2 were both elevated in vessels subjected to the intravascular imaging procedure (Figure S1D,E). Importantly, there were no differences in plaque area between groups (Figure S1F).

Comparison of vessel and plaque features between grafted thoracic aortas collected at the 6‐week midpoint and the final 12‐week timepoint revealed there were no differences in plaque area and outer vessel area (Figure S2A,B). However, there was an increase in lumen area from the midpoint to the final timepoint, a sign of positive remodeling that occurs with disease progression (Figure S2C, *p *< 0.001), which was accompanied by a modest reduction in the degree of stenosis at the final timepoint (Figure S2D, *p *< 0.01).

### 2.2 OCT‐Acquired Plaque Lipid Assessment Correlates With Histologically Acquired Plaque Lipid Content

Signal‐poor regions with diffuse borders in OCT images indicate the presence of plaque lipid, a key feature of unstable atherosclerotic lesions [22]. To estimate plaque lipid distribution, the optical attenuation at each angle (A‐line) in the OCT images was calculated using a published algorithm [22, 23, 24] and displayed as pseudo‐colored rings surrounding the OCT grayscale image showing high (red) to low (blue) lipid accumulation (Figure 2A,C). Ex vivo Oil Red O (ORO) staining showed that these lipid‐rich areas detected by OCT corresponded to lipid‐rich areas in plaques from co‐registered sections (Figure 2B,D). To better visualize the lipid plaque distribution, a 2D OCT attenuation map (Figure 2E) was created, where blue and black lines indicate the position in the pullback scan where Figure 2A,C were acquired. Comparison of plaque ORO % with OCT attenuation % demonstrated corresponding values that spanned every 200 µm across the entire artery (Figure 2F). Furthermore, assessment of plaque ORO % and OCT attenuation % for all mice in this study revealed a positive correlation between these two lipid assessment methods (R^2^ = 0.199, *p *< 0.001, Figure 3).

**FIGURE 2:**
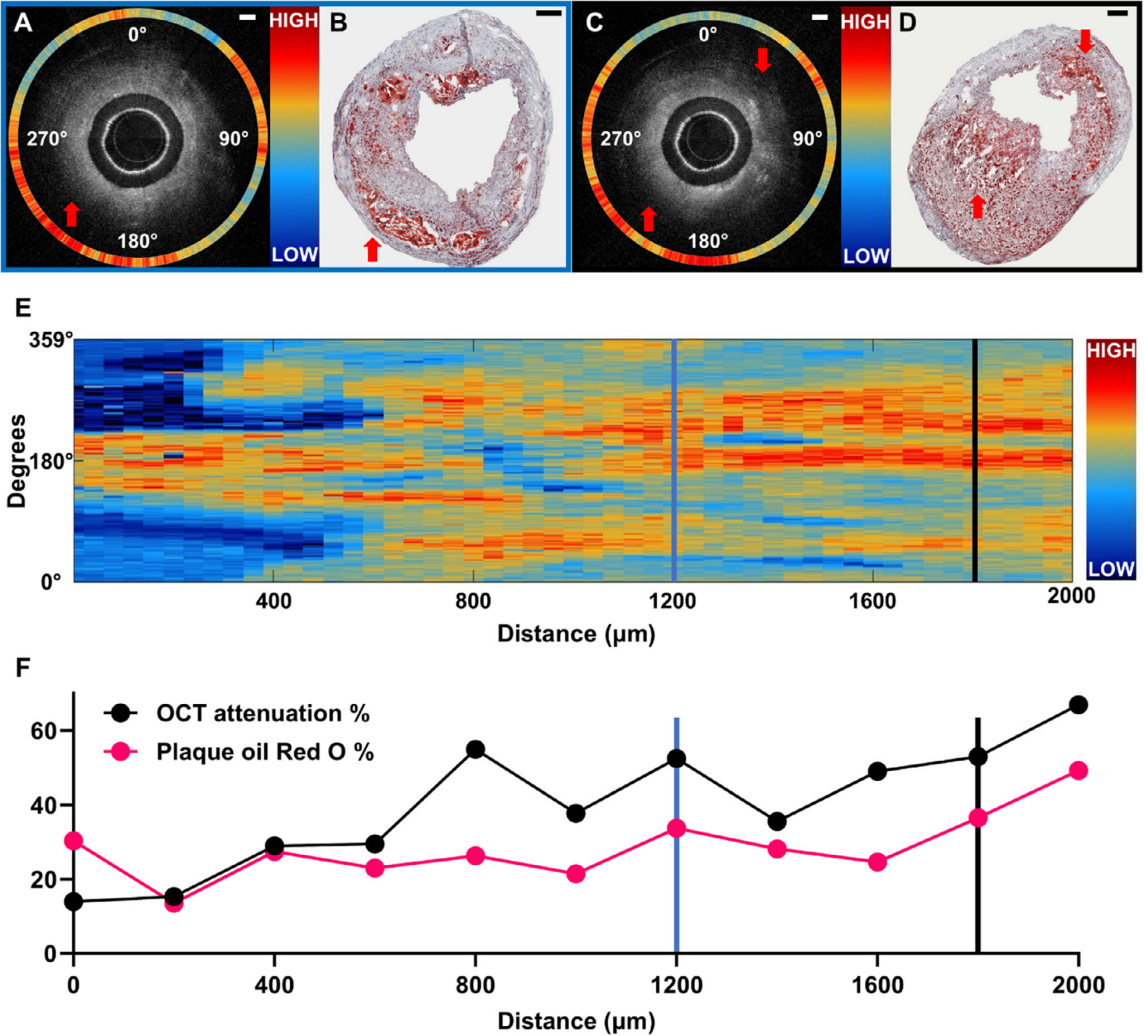
OCT‐acquired plaque lipid assessment correlates with histologically acquired plaque lipid content. (A, C) Representative OCT images depicting lipid‐rich plaques as signal‐poor regions with diffuse borders (red arrows). Optical attenuation was calculated and displayed as pseudo‐colored rings surrounding the OCT greyscale image showing high (red) to low (blue) lipid content. (B, D) Corresponding cross‐sectional images depicting Oil Red O‐stained plaque lipid. (E) *En face* OCT attenuation map generated by an attenuation calculation showing high (red) to low (blue) lipid accumulation along the length of the vessel. Blue and black lines show where Figure 2A,C were acquired. (F) Comparison of the distribution of lipid percentages along the length of the vessel between in vivo OCT attenuation % and ex vivo plaque Oil Red O % assessments (*n* = 1). Scale bars: 100 µm.

**FIGURE 3:**
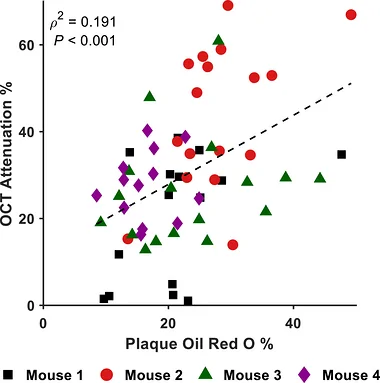
OCT attenuation correlates with plaque Oil Red O content. Scatter plot of OCT attenuation % vs. Plaque Oil Red O %. Each symbol represents values spanning every 200 µm across the entire artery of an individual mouse (*n* = 4). Spearman's correlation coefficient (ρ^2^) and *p*‐value (*P*) are shown.

### 2.3 NIRF Acquired Using Dual Modality Imaging Probe Correlates With Histological Plaque Macrophage Measures

Previous studies show that enhanced ICG fluorescence signals co‐localize with macrophage‐abundant atheroma [14, 20]. This is particularly evident in advanced high‐risk plaques with macrophage‐rich cores and plaque inflammation. For this study, fluorescence signals were displayed as a pseudo‐colored fluorescent intensity scale with high (red) to low (blue) fluorescence intensity values. We found that fluorescence values calculated from NIRF images could differentiate between macrophage‐poor and macrophage‐rich plaques. We see low NIRF signal (Figure 4A) in plaques with lower levels of CD68^+^ macrophage immunofluorescent staining (Figure 4B). Conversely, a higher NIRF signal (Figure 4C) is evident in plaques with more CD68^+^ immunostaining (Figure 4D). Reconstruction of a 2D *en face* NIRF signal map (Figure 4E) highlighted NIRF‐positive regions spanning the entire artery, with the blue and red lines representing macrophage‐poor (matched with Figure 4A,B) and macrophage‐rich (matched with Figure 4C,D) regions, respectively.

**FIGURE 4:**
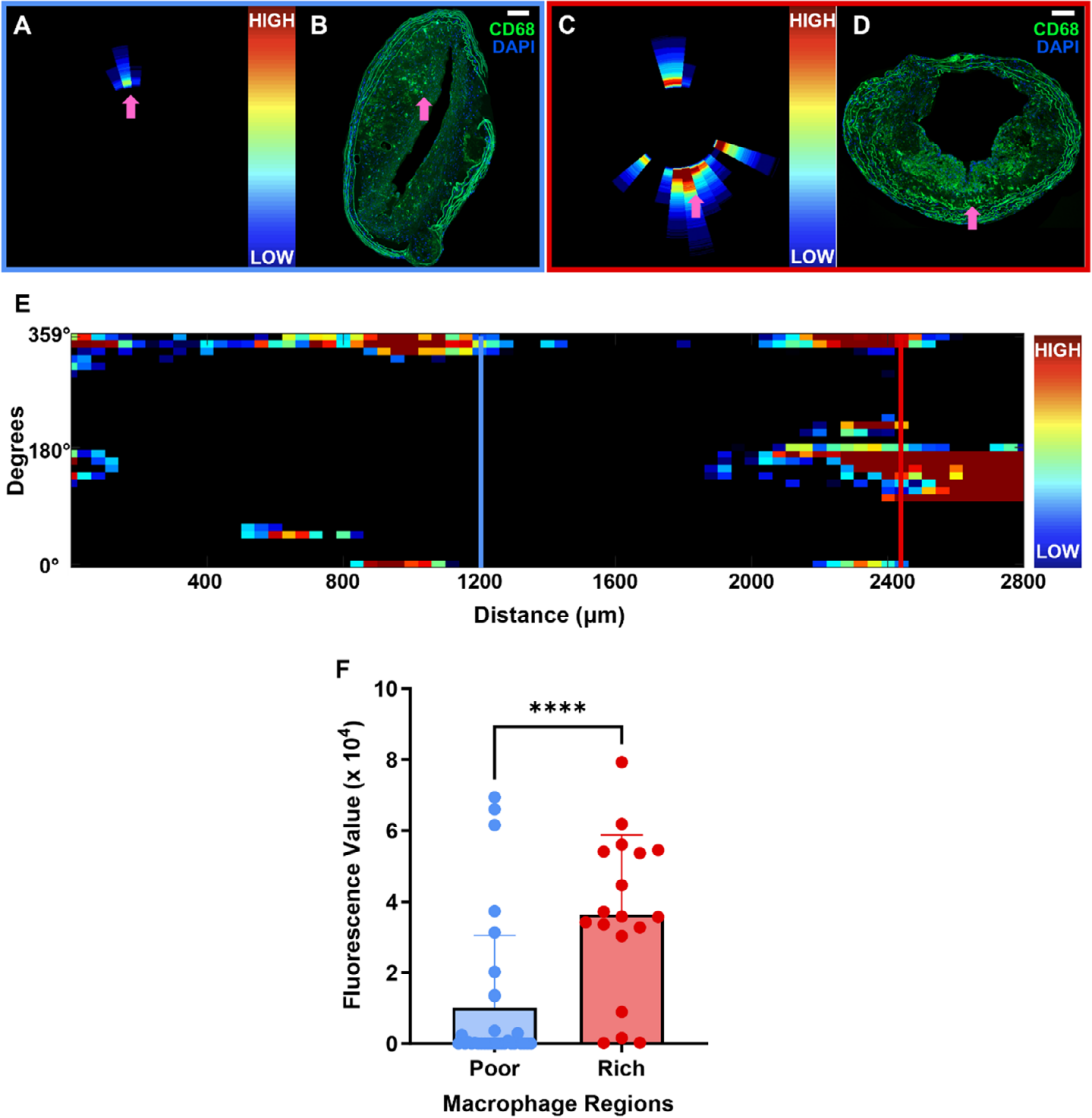
NIRF acquired using dual modality imaging probe correlates with histological plaque macrophage measures. (A, C) Representative fluorescence images taken at Week 12. Pseudo‐colored fluorescence intensity scale showing high (red) to low (blue) ICG fluorescence. (B, D) Corresponding images of plaque cross sections immunofluorescently assessed for CD68^+^ macrophages (green staining, pink arrow) at Week 12. (E) *En face* NIRF map, showing high (red) to low (blue) NIRF signal, indicating colocalization with macrophages. Blue and red lines show where Figure 4A,C were acquired. (F) Fluorescence (NIRF) values from co‐registered plaque sections that were immunofluorescently assessed as macrophage‐poor (<50% CD68^+^ staining) and macrophage‐rich (>50% CD68^+^ staining). The radial axis of the fluorescence images (A, C) represents wavelength, not tissue depth, and provides a color‐coded representation of the spectrum acquired at each orientation. Data are expressed as Mean±SD. ^****^ *p *< 0.0001 by Mann–Whitney test, (*n* = 4). Scale bars: 100 µm.

Consistent with this, we next stratified all CD68^+^ immunostained plaque sections into macrophage poor (<50% plaque CD68^+^) and macrophage rich (≥50% plaque CD68^+^). This demonstrated that the NIRF signal value was significantly higher (3.5 ± 2.2 × 10^4^) in co‐registered plaque sections that were macrophage rich, when compared to the NIRF signal value (0.7 ± 1.8 × 10^4^) from co‐registered plaque sections that were macrophage poor (Figure 4F, *p *< 0.0001).

Interestingly, comparison of NIRF signal values acquired from the middle and final imaging timepoints (Figure 5A,B) found that the NIRF signal was higher at the middle timepoint (152.9 ± 77.8 × 10^4^) compared to the final timepoint (2.0 ± 2.5 × 10^4^, *p *< 0.0001) (Figure 5C). Supporting this, plaque CD68^+^ macrophage content (Figure 5D,E) was also higher in plaque sections collected from the middle timepoint (62.2 ± 12.1%) compared to the final timepoint (40.9 ± 24.1%, *p *< 0.001) (Figure 5F).

**FIGURE 5:**
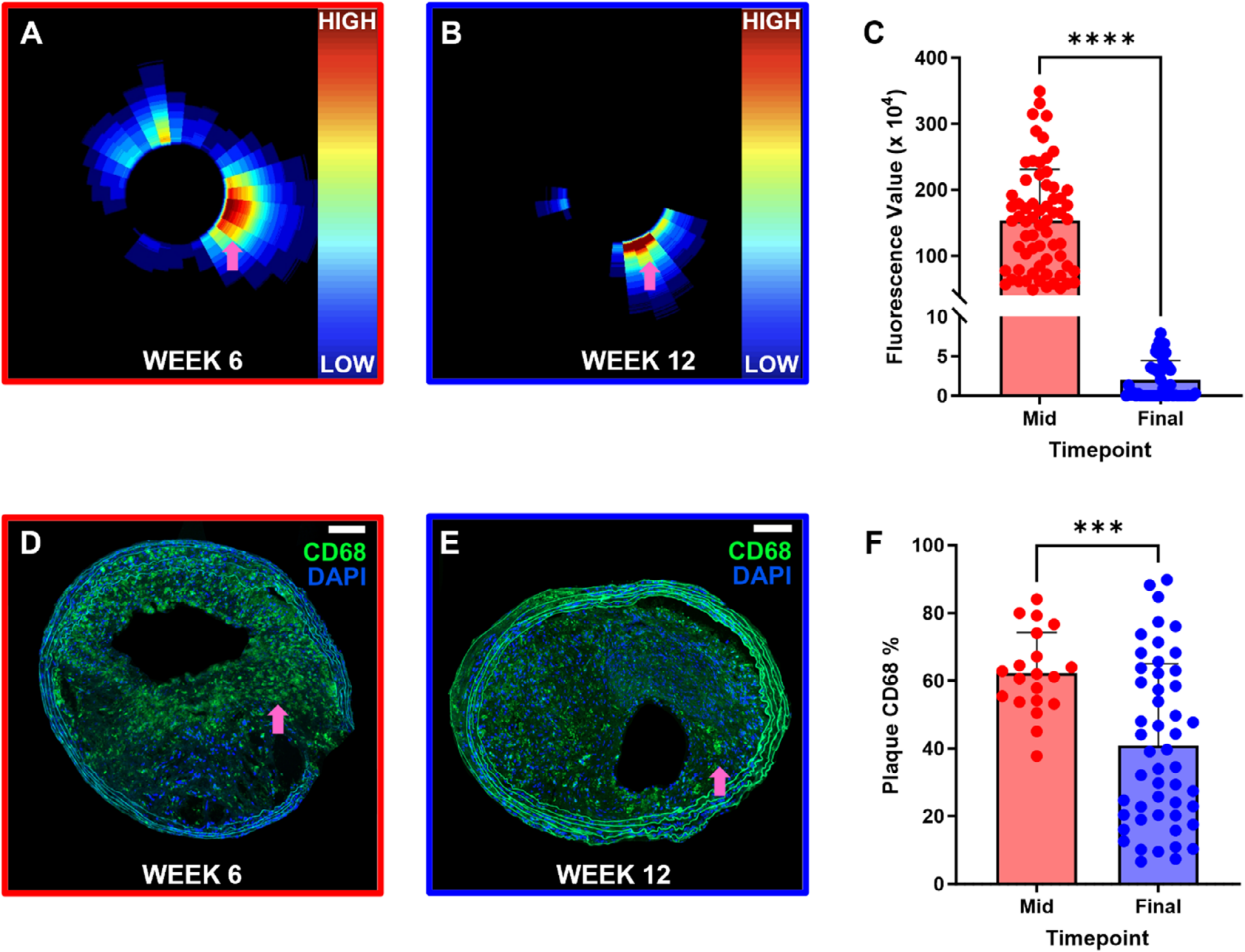
NIRF signal and macrophage content is higher at the middle timepoint than the final timepoint. Representative fluorescence images taken at (A) Week 6 middle imaging timepoint and (B) Week 12 final imaging timepoint. Pseudo‐colored fluorescent intensity scale showing high (red) to low (blue) NIRF, indicating macrophage colocalization. (C) NIRF values of middle and final imaging timepoints. Images of co‐registered plaque cross sections immunofluorescently assessed for CD68^+^ macrophages (green staining, pink arrows) from (D) Week 6 middle timepoint and (E) Week 12 final timepoint. (F) Plaque CD68^+^ % analysis of middle and final timepoint plaque sections. The radial axis of the fluorescence images (A, B) represents wavelength, not tissue depth, and provides a color‐coded representation of the spectrum acquired at each orientation. Data expressed as Mean±SD. ^***^ *p *< 0.001, ^****^ *p *< 0.0001 by Mann–Whitney test, (*n* = 4). Scale bars: 100 µm.

We found significant correlations between OCT attenuation and plaque ORO staining (Figure S3A, ρ^2^ = 0.191, *p* < 0.001), and OCT attenuation and plaque CD68+ immunofluorescence (Figure S3B, ρ^2^ = 0.116, *p* = 0.007). There was a weak correlation between ICG fluorescence and plaque ORO staining (Figure S3C, ρ^2^ = 0.015, *p* = 0.341) and a slightly stronger correlation between ICG fluorescence and plaque CD68+ fluorescence (Figure S3D, ρ^2^ = 0.452, *p* < 0.001).

## 3 Discussion

Longitudinally capturing the transformation of a stable atherosclerotic plaque into a high‐risk, life‐threatening lesion or visualizing the effects of therapeutic interventions has the potential to reshape clinical strategies for managing coronary artery disease. The overarching objective of the present study was to demonstrate comprehensive longitudinal intravascular assessment of atherosclerotic plaque development across the entirety of a diseased vessel in mice. This was achieved using a murine surgical carotid interposition grafting model and miniaturized OCT+fluorescence imaging probe. We now report the following important findings: (1) OCT attenuation that assesses plaque lipid arc via our imaging probe closely correlated with histological ORO examination of plaque lipid in co‐registered sections spanning the aortas, (2) ICG fluorescence (NIRF), captured by the imaging probe, also correlated positively with histologically assessed macrophage rich plaques, both declining in value over time suggesting stabilization, and (3) lipid and NIRF data acquired by the probe can be rendered to generate *en face* and 3D images of plaque distribution along the length of the vessels. These findings demonstrate the successful development of a novel approach for longitudinal assessments of atherosclerosis of entire vessels. Our model has significant advantages over histological assessments which are limited to a single timepoint per mouse and are unable to fully capture atherosclerotic disease throughout a vessel or changes in stability over time.

Utilizing the murine carotid interposition grafting surgical procedure, we place the larger thoracic aorta (∼1.2 mm inner diameter), which is comparable in size to some smaller human coronary arteries, from a donor mouse into the carotid artery region of a recipient mouse. This enables insertion of miniaturized intravascular imaging probes, such as ours (outer diameter 0.635 mm), that would otherwise not fit inside murine carotid arteries without causing endothelial damage, stretching or tearing. Another advantage of grafting the thoracic aorta into the carotid region is that it provides the most accessible site for intravascular probe insertion. By creating a small arteriotomy at the base of the grafted thoracic aorta, probe access into the graft at multiple timepoints facilitates longitudinal assessment of disease. Before insertion of the probe and arteriotomy placement, the grafted thoracic aorta is clamped at either end on top of plastic cuffs used in the grafting procedure. The need to clamp the vessel before intravascular imaging makes grafting into the carotid region essential so blood flow to the brain can be maintained, and therefore survival, via the other, left carotid artery. Mice would not survive intravascular probe insertion directly into the thoracic aorta, due to it impeding critical blood flow. Overall, our grafting method with arteriotomy is a unique approach that enables longitudinal intravascular imaging of murine atherosclerosis that no other murine model could achieve.

The inclusion of a non‐imaged control cohort in our study enabled direct assessment of the vascular impact of the intravascular imaging procedure. The results of the control cohort indicate that intravascular imaging is largely well tolerated in this model and does not significantly perturb plaque structure or vessel integrity, although evidence of minor inflammatory activation was present. All experimental studies using this model should include appropriate vehicle or genetic control groups to account for biological responses induced by the imaging procedure.

The ability to perform these measures in mice, rather than larger rodent or porcine models, has distinct biological and practical advantages for assessing atherosclerosis. Housing and breeding mice are cost‐effective, and their size allows for smaller quantities of drug treatments or gene therapies to be used. Mechanistic assessments are possible using gene deletion, overexpression or fluorescent labelling of specific cells or proteins that can be tracked. Furthermore, the stages of atherosclerosis development when on HCD are well‐characterized so interventions can be robustly tested at both early and established plaque development stages. Finally, there is excellent availability of antibodies, enzyme‐linked immunosorbent assays, and other enzymatic or colorimetric assays for mice that are more limited for other species.

Our model is distinctly different to intravital imaging [25]. Intravital imaging captures vessel information from the outside in. Whilst it can measure plaque changes serially in the carotid aorta, the accuracy of this is limited as it only captures the area of plaque visualized through the transparent vessel, and it does not provide deeper plaque information. By contrast, our model, through intravascular access, enables volumetric plaque measures, combined with the distribution of inflammation through the plaque that is able to be visualized in three dimensions. The strength of intravital imaging is the ability to observe monocyte, neutrophil, and other immune cell trafficking, which is unable to be measured in our model. Intravital imaging has technical challenges that include the requirement to stabilize the pulsatile carotid vessel, including bright reporters or molecular probes, and for two‐photon or high‐speed confocal/intravital microscopes to manage the motion and depth limits. It is possible for our model to be combined with intravital imaging that would offer the strengths of each.

Previously, attempts were made to image mice arteries by utilizing an OCT‐only clinically available catheter [26]. Although neither longitudinal nor OCT+fluorescence multimodal imaging was achieved by Tahara et al., this previous attempt highlighted the significance of intravascular imaging in mice. Clinically available intravascular imaging techniques mostly describe gross plaque morphology and do not capture important compositional features that are critically linked to future risk [11, 20, 27]. Our highly miniaturized OCT+fluorescence multimodal imaging catheter simultaneously measures both structural and compositional information, providing high‐resolution 3D imaging capabilities in vivo across the entire diseased vessel. This dual modality imaging catheter utilized a double‐clad fiber, where the core of the fiber collects OCT signal while the inner cladding collects the fluorescence signal, as demonstrated in our previous work [28, 29, 30]. In our imaging catheter, a thin‐walled coil (with an outer diameter of 0.36 mm, to transfer the rotation from the proximal to the distal end) was incorporated [16] to ensure the outer diameter of the device (0.635 mm) fits within the grafted aorta. The advantage of our longitudinal murine imaging platform is that it can be used to monitor the evolution of high‐risk plaque characteristics over time, with co‐located biological (molecular) and microstructural information to track large lipid cores, cholesterol crystals, macrophage‐rich regions and thin fibrous caps. Consistent with previous studies [22, 23, 24], we demonstrated that plaque lipid content can be evaluated by OCT using our attenuation algorithm to provide the spatial location of lipid within the vessel. These measurements correlated with *ex vivo* histopathological analyses of plaque lipid content by ORO staining. Furthermore, our biochemical NIRF imaging showed high ICG fluorescence signal, indicating macrophage‐rich regions, which was confirmed by CD68^+^ immunofluorescence staining. These correlations are consistent with previous studies performed in pigs and humans [14, 20].

The ability to longitudinally image plaque in the same vessel in the same mouse is an important capability of our mouse model. The mouse can act as its own baseline control, which reduces inter‐animal variations in disease development and in response to treatments. The intravascular imaging enabled by our model can also capture plaque throughout an entire vessel in 3D. Together, this approach significantly improves the precision and power of the plaque analyses, meaning less mice are required to observe the effects of an intervention. Our model represents a paradigm shift over the current need to compare disparate plaques histologically across multiple timepoints and multiple animals. Histological assessments are therefore unable to fully capture plaque development, even with detailed systematic analysis of numerous tissue sections per vessel, so regions of plaque information are lost. Histology is also labor‐intensive and dependent on antibody performance.

## 4 Study Limitations

Despite the number of innovations included in this study, several limitations exist. The first is the high‐level of skill and training required for the murine surgical model. The tools and equipment needed to conduct the model are not, however, expensive or specialized. It is acknowledged that the grafting surgical procedure may influence plaque development. Our analyses of grafted vessels showed typical robust development of macrophage‐rich atherosclerotic plaques. Furthermore, all experimental studies using this model should include appropriate vehicle or genetic controls. Finally, the miniaturized multi‐modal intravascular imaging device is custom‐built, so it may be challenging to replicate this aspect of the work, but other small diameter imaging devices could also be applied successfully to our murine model.

## 5 Conclusions

We have developed and validated an innovative research platform for longitudinal intravascular assessments of plaque development in mice. Our miniaturized multimodal imaging probe captures high‐resolution images showing clinically relevant plaque features while simultaneously measuring fluorescence from ICG. Assessing plaque intravascularly has the power to generate 3D images of the entire diseased vessel, a significant advantage over histological approaches. Murine intravascular imaging that is performed in a longitudinal manner improves the ability to accurately assess the effect of therapeutic interventions on plaque development. Our model provides an important new addition to the translational research pipeline for the assessment of atherosclerosis.

## 6 Experimental Section

### 6.1 Murine Carotid Interposition Grafting Model

All experimental procedures were conducted with approval from the South Australian Health and Medical Research Institute Animal Ethics Committee (SAM 425.19) and conformed to the Guide for the Care and Use of Laboratory Animals (United States National Institute of Health). To enable in vivo intravascular imaging, the carotid interposition grafting mouse model was utilized as described previously [15].

Eight‐week‐old male *Apoe* ^−/−^ donor mice were fed a HCD containing 21% fat and 0.15% cholesterol (SF‐00219, Specialty Feeds, Glen Forrest, WA, Australia) for 6 months to promote plaque development in the thoracic aorta. After euthanasia, the thoracic aorta (∼10 mm in length) from the donor mouse was harvested using electrocautery. Eight‐week‐old male *Apoe* ^−/−^ recipient mice were fed a HCD for 3 months prior to surgery. A ventral midline incision (4–5 mm) was made in the neck to expose the right common carotid artery. Two sutures were tied around the artery, which was then cut at the midpoint. Polyethylene cuffs (0.6 mm in diameter) were placed over the ends of the carotid artery and anchored with clamps. The carotid artery ends were everted over the cuffs and secured with 8‐0 silk sutures. The donor thoracic aorta was then sleeved over the cuffs, secured with 8–0 silk sutures, and clamps were removed.

Following the grafting surgery, mice (*n* = 4) were fed HCD for a further 12 weeks in total. In vivo intravascular imaging was performed at surgery (baseline) and then at weeks 6 and 12 post‐grafting.

### 6.2 OCT and Fluorescence Intravascular Imaging System

We have developed an OCT+fluorescence imaging system [16], where a double‐clad fiber coupler (Castor Optics Inc., Canada) is used to integrate a 1300 nm OCT system (Telesto II, Thorlabs GmbH, Germany) and a spectrometer‐based NIRF system (Figure S4). The spectrometer (QE Pro, Ocean Optics, USA) acquires fluorescence signals over a 356–1123 nm wavelength range, enabling spectral unmixing to extract the ICG fluorescence signal from background autofluorescence and reflection artifacts. The intravascular imaging probe was rotated at 1 revolution per second by a counter‐rotation motor and pulled back at approximately 40 µm/s by a motorized linear stage.

### 6.3 In Vivo OCT+Fluorescence Imaging Procedure

One hour prior to imaging, ICG, an FDA‐approved near‐infrared fluorescent agent, was injected intravenously (0.5 mg/kg, 130 µg/ml solution). ICG is an established NIRF marker that indicates plaque permeability and colocalizes with macrophages [20]. Mice were placed under anesthesia, and the grafted aorta was isolated, flushed, and clamped at the proximal and distal ends with cuffs. A small transverse arteriotomy was made in the graft at the proximal end into which the OCT+fluorescence catheter was inserted, and intravascular imaging was performed. Following imaging, a cotton tip was held on the entrance site (away from the main analysis site) before suturing the arteriotomy closed with interrupted sutures (10–0 suture), then the skin (6–0 suture), and the mouse was allowed to recover.

At the final imaging timepoint, after imaging, the grafted arteries were saline perfused and excised for *ex vivo* histopathological analyses.

### 6.4 OCT+Fluorescence Image Analysis

Based on the single scattering model [22, 23, 24], the optical attenuation coefficients (OAC) of each A‐line in OCT images were calculated using our algorithm developed in MATLAB 2021b (Mathworks Inc., Natick, MA, USA). The algorithm was designed to automatically find edges of the lumen and the end of regions of interest, then apply least‐squares linear fitting to the log‐compressed data to calculate the OAC. The extracted OACs were then color‐coded on a linear scale between 0 and 22 mm^−1^ and displayed around the OCT image. Figure S5 depicts the result of the automatic edge finding, the calculated OACs of a certain OCT frame, and the final parametric imaging of OAC with the OCT image.

To be comparable with ORO results, we defined PL_OAC_ as the lipid percentage calculated from OAC.

(1) $$P\;L_{OAC}=\frac{N\left(\mu_{t}>T_{OAC}\right)}{N_{A-line}}\;\times 100\%$$

N is the number of A‐lines, µ_t_ is OAC, T_OAC_, which is set to 13 mm^−1^ in this study, is the OAC threshold to be considered as lipid plaque [22], N_A‐line_ is the total number of A‐lines in each OCT frame.

Acquired NIRF signals within the 750–950 nm wavelength range were automatically processed using a least‐squares linear spectral unmixing algorithm [31] to separate the ICG fluorescence signal from background autofluorescence and reflection artifacts. Representative ICG and background spectra, the spectral components used for unmixing, and detailed comparisons between background/reflection and ICG spectra are presented in Figure S6. The processed signal was then color‐coded linearly across its intensity range and overlaid with the OCT image. The fluorescence spectrum was visualized as a color‐coded ring, with shorter wavelengths (starting at 800 nm, the cutoff of the long‐pass filter) displayed on the inner side of the ring and longer wavelengths on the outer side, then either color coded linearly across its value range to be displayed or summed along each A‐line to generate a single value for further analysis.

### 6.5 Ex Vivo Histopathology

Arteries were embedded in Tissue‐Tek OCT compound (IA018, Sakura Finetek, Japan) and stored at −80°C. Serial cryosections (5 µm) were collected from the entire grafted artery. Four consecutive cryosections were collected per slide (equivalent to a single OCT+fluorescence frame of 20 µm). Histological analyses performed on cryosections (200 µm apart/15–20 per animal) include: Hematoxylin and eosin (H&E, plaque area), ORO (lipid), and CD68^+^ immunofluorescence (macrophages, MCA1957GA, Bio‐Rad, Hercules, CA, USA). Images were analyzed using Image‐Pro Premier software (Version 9.2, Media Cybernetics, Rockville, MD, USA).

OCT+fluorescence imaging data were matched to co‐registered histological sections using structural landmarks (e.g., exit point of imaging catheter and side branches) and specific plaque features as well as the known location of collection along the vessel.

### 6.6 Statistics

Data are expressed as Mean±SD. Normal distribution of data was determined using the Shapiro–Wilk test. Differences between groups were calculated using Mann–Whitney test. Significance was set at a two‐sided *p *< 0.05.

## Author Contributions

**Lei Xiang**: software, formal analysis, visualization, Writing – review and editing. **Jiawen Li**: conceptualization, data curation, formal analysis, visualization, writing – original draft, methodology, investigation, supervision, writing – review and editing, funding acquisition, resources. **Jiandi Liu**: writing – review and editing, data curation, formal analysis. **Joanne T. M. Tan**: formal analysis, writing – review and editing. **Ayla Hoogendoorn**: conceptualization, methodology, funding acquisition, writing – review and editing. **Johan W. Verjans**: conceptualization, funding acquisition, writing – review and editing. **Richard Le**: writing – review and editing, data curation. **Lauren Sandeman**: writing – review and editing, data curation. **Victoria A. Nankivell**: writing – review and editing, data curation. **Peter J. Psaltis**: funding acquisition, writing – review and editing. **Rouyan Chen**: writing – review and editing, data curation, formal analysis. **Christina A. Bursill**: resources, funding acquisition, conceptualization, writing – original draft, supervision, project administration, writing – review and editing. **Robert A. Mclaughlin**: writing – review and editing, resources, funding acquisition, conceptualization.

## Funding

ARC Centre of Excellence for Nanoscale BioPhotonics, (CE140100003), National Health and Medical Research Council (NHMRC) Ideas Grant 2001646 (J.L., J.W.V., R.A.M., P.J.P., C.A.B.). NHMRC Development grant 2022337 (J.L., P.J.P., R.A.M.). NHMRC Investigator Grant 2008462 (J.L.). Heart Foundation Future Leader Fellowship 105608 (J.L.). Hospital Research Foundation project grant 2022‐CP‐IDMH‐014‐83100 (J.L., P.J.P., J.W.V., R.A.M.). NHMRC Ideas grant 1184571 (C.A.B., P.J.P.). Lin Huddleston Heart Foundation Fellowship (C.A.B.).

## Conflicts of Interest

J.L. is the founder and Director of Theia Medical Pty Ltd, a company that develops medical devices but did not contribute to this study. R.A.M. is director of Miniprobes Pty Ltd. that develops optical imaging systems, but did not contribute to this study. P.J.P. has received research support from Abbott Vascular, consulting fees from Amgen and Esperion, and speaker honoraria from AstraZeneca, Bayer, Boehringer Ingelheim, Merck Schering‐Plough, and Pfizer. J.W.V. is director of Caremappr Pty Ltd but did not contribute to this study; he received research support from Medtronic, Roche, Amgen, Sanofi, and received honoraria/speaking fees from Sanofi, Eli Lilly, Novartis, Boehringer Ingelheim.

## Supporting information




**Supporting File 1**: advs77316‐sup‐0001‐SuppMat.docx.

**Supporting File 2**: advs77316‐sup‐0002‐MovieS1.mp4.

## Data Availability

The data that support the findings of this study are available from the corresponding author upon reasonable request.
