# Supplementary material for: Serial Molecular‐Microstructural Multimodal Intravascular Imaging of Atherosclerotic Plaques in Mice
Source: Adv Sci (Weinh). 2026 Aug 19;13(62):e77316. doi: 10.1002/advs.77316 (PMC13488478; doi:10.1002/advs.77316)
Supplement: Supplementary file 1 [file ADVS-13-e77316-s001.docx]

Supporting Information

Serial molecular-microstructural multimodal intravascular imaging of atherosclerotic plaques in mice

**Authors:** Jiawen Li, Joanne T. M. Tan, Lei Xiang, Jiandi Liu, Ayla Hoogendoorn, Victoria A. Nankivell, Lauren Sandeman, Richard Le, Rouyan Chen, Robert A. McLaughlin, Peter J. Psaltis, Johan W. Verjans, Christina A. Bursill

From the Discipline of Biomedical Engineering, School of Electrical and Mechanical Engineering, Adelaide University (JW.L., L.X., R.C.), Institute for Photonics and Advanced Sensing, Adelaide University, Adelaide (JW.L, R.C., L.X., R.A.M.), Australian Research Council (ARC) Centre of Excellence for Nanoscale BioPhotonics (JW.L., R.A.M., C.A.B.), Vascular Research Centre, Lifelong Health Theme, South Australian Health and Medical Research Institute (SAHMRI) (JW.L., J.T.M.T., JD.L., A.H., V.A.N., L.S., R.L., R.C., P.J.P., J.W.V., C.A.B.), Adelaide Medical School, Adelaide University (J.T.M.T., V.A.N, P.J.P., J.W.V., C.A.B.), Flinders Medical Centre (R.L.), Department of Cardiology, Second Affiliated Hospital, Jiangxi Medical College, Nanchang University (JD.L.), School of Biomedicine, Adelaide University (R.A.M.) Department of Cardiology, Royal Adelaide Hospital (P.J.P, J.W.V.)

**Correspondence to:**

A/Prof Jiawen Li and Prof Christina Bursill

Vascular Research Centre, SAHMRI,

Adelaide University.

North Terrace, Adelaide, 5000, South Australia, Australia

**Emails:**	jiawen.li01@adelaide.edu.au and Christina.Bursill@sahmri.com

**Work Telephone Number:**	+61 881 284 788

**Supplementary Figure S1**

**Supplementary Figure S1. Effect of inserting the intravascular imaging catheter on plaque characteristics.** Apolipoprotein (*Apo*)*e*^-/-^ donor mice were fed HCD for 6 months. The thoracic aorta from the donor mouse was carotid interposition-grafted into *Apoe^-/-^* recipient mice fed HCD for 3 months prior to surgery. For the ‘control’ group no intravascular imaging was performed (N=3). For the ‘intervention’ group intravascular imaging of the grafted aorta was performed at surgery (baseline) and then 6- and 12-weeks post-surgery (N=4). Vessel sections spanning the grafted thoracic aortas from each group were histologically assessed for **(A)** CD31^+^ endothelial cells, **(B)** αSMA+ smooth muscle cells, **(C)** VCAM1^+^, **(D)** iNOS, **(E)** CCL2 and **(F)** plaque area. Data presented as mean±SD. Each data point on bar graphs represents a section. Two groups were compared using Welch’s t tests for parametric data or the Mann–Whitney U test for nonparametric data. **P*<0.05, ****P*<0.0001. αSMA: alpha smooth muscle cell actin; VCAM1: vascular cell adhesion molecule 1; iNOS: inducible nitric oxide synthase; CCL2: CC chemokine ligand 2.

**Supplementary Figure S2**

**Supplementary Figure S2. Plaque and vessel measures of the 6-week midpoint versus the 12-week final time point grafted thoracic aortas.** Apolipoprotein (*Apo*)*e*^-/-^ donor mice were fed HCD for 6 months. The thoracic aorta from the donor mouse was carotid interposition-grafted into *Apoe^-/-^* recipient mice fed HCD for 3 months prior to surgery. Intravascular imaging of the grafted aorta was performed at surgery (baseline) and then 6-weeks (midpoint) and 12-weeks (final) post-surgery (N=4). Thoracic aorta sections spanning grafted vessels from the 6-week mid-point and the 12-week final time point were assessed for **(A)** Plaque area**, (B)** Vessel area**, (C)** Lumen area and **(D)** %Stenosis. Data presented as mean±SD. Each data point on bar graphs represents a section. Two groups were compared using Welch’s t tests for parametric data or the Mann–Whitney U test for nonparametric data. ***P*<0.01, ****P*<0.0001

**Supplementary Figure S3**

**Supplementary Figure S3. Spearman’s Correlations between imaging and histological data. (A)** Correlation between OCT attenuation and plaque lipid quantified using Oil Red O (ORO) staining. **(B)** Correlation between OCT attenuation and plaque macrophages quantified using CD68 immunofluorescence histology. **(C)** Correlation between ICG fluorescence and plaque lipid quantified using ORO staining. **(D)** Correlation between ICG fluorescence and plaque macrophages quantified using CD68 immunofluorescence histology. OCT: optical coherence tomography.

**Supplementary Figure S4**

**Supplementary Figure S4. Optical coherence tomography (OCT) + fluorescence intravascular system and double-clad fiber (DCF)-based probe for serial imaging in mice.** Using a customized double-clad fiber (DCF) coupler, OCT and 785nm fluorescence excitation light was combined into the same DCF that delivered and collected light inside the mice, specifically the grafted aorta. OCT signal was collected from a spectrometer of a spectral domain OCT (Telesto II, Thorlabs GmbH, Germany). The fluorescence signal collected from a portable spectrometer (QE Pro, Ocean Optics, USA). Both signals were processed and displayed in a laptop workstation. WDM: wavelength division multiplexer. Blue line: single mode fiber; orange line: multimode fiber; yellow line: double clad fiber; black line: cables for electrical signals.

**Supplementary Figure S5**

**Supplementary Figure S5. Calculation of Optical Attenuation Coefficient (OAC) for determination of atherosclerotic plaque lipid content.** (**A**) Original optical coherence tomography (OCT) image. (**B**) Automatic edge finding result for the OAC calculation area, green line: calculation start point, red line: calculation end point. (**C**) Calculated OAC. (**D**) Color-coded OAC value displayed around OCT image as depicted in Figure 2C with a spectrum of high (red) and low (blue) signals.

**Supplementary Figure S6. Fluorescence spectral unmixing process and components analysis.** (**A**) Optical Coherence Tomography (OCT) image. (**B**) Original measured fluorescence image. (**C**) Background-subtracted fluorescence image. (**D**) Spectral unmixed fluorescence image. (**E**) Background autofluorescence component and Indocyanine Green (ICG) fluorescence component for spectral unmixing. (**F**) Original measured spectra of external reflection (RF) and ICG. (**G**) Background-subtracted spectra of RF and ICG. (**H**) Unmixed spectra of RF and ICG. (**I**) Comparison of the background autofluorescence component and external reflection spectrum. (**J**) Background autofluorescence component and ICG component weighting factors across the entire image. Note that the fluorescence images (**B, C, D**) graphically represent the spectrum measured at each orientation of the rotating probe. Distance from the center of the image (i.e. radial position) indicates wavelength (not depth in tissue) and intensity at each wavelength is color-coded. Green dashed lines indicate the A-scan position of the external reflection; red dashed lines indicate the A-scan position of the ICG. $C_{BG}$, background autofluorescence component; $C_{ICG}$, ICG fluorescence component; RF, external reflection; ICG, Indocyanine Green fluorescence; $\beta_{BG}$, background autofluorescence component weighting factor; $\beta_{ICG}$, ICG component weighting factor; r, correlation coefficient; p, probability value. This fluorescence signal processing algorithm has been reported in our 2025 publication on PNAS Nexus (Reference 32 of the main manuscript), where we also shared this in vivo mouse imaging dataset via Figshare (https://doi.org/10.25909/29595041).
